# Fibrocytes are increased in lung and peripheral blood of patients with idiopathic pulmonary fibrosis

**DOI:** 10.1186/s12931-018-0798-8

**Published:** 2018-05-10

**Authors:** P. Heukels, J. A. C. van Hulst, M. van Nimwegen, C. E. Boorsma, B. N. Melgert, L. M. van den Toorn, K. A. T. Boomars, M. S. Wijsenbeek, H. Hoogsteden, J. H. von der Thüsen, R. W. Hendriks, M. Kool, B. van den Blink

**Affiliations:** 1 Department of Pulmonary Medicine, Erasmus MC, s-Gravendijkwal 230, 3015 CE Rotterdam, The Netherlands; 2 Department of Pharmacokinetics, Toxicology and Targeting, Groningen Research Institute for Pharmacy, University of Groningen, Groningen, The Netherlands

**Keywords:** Fibrocytes, Idiopathic pulmonary fibrosis, Lung Fibrocytes, Pulmonary hypertension, Flow cytometry

## Abstract

**Background:**

Fibrocytes are implicated in Idiopathic Pulmonary Fibrosis (IPF) pathogenesis and increased proportions in the circulation are associated with poor prognosis. Upon tissue injury, fibrocytes migrate to the affected organ. In IPF patients, circulating fibrocytes are increased especially during exacerbations, however fibrocytes in the lungs have not been examined.

Therefore, we sought to evaluate if fibrocytes can be detected in IPF lungs and we compare percentages and phenotypic characteristics of lung fibrocytes with circulating fibrocytes in IPF.

**Methods:**

First we optimized flow cytometric detection circulating fibrocytes using a unique combination of intra- and extra-cellular markers to establish a solid gating strategy. Next we analyzed lung fibrocytes in single cell suspensions of explanted IPF and control lungs and compared characteristics and numbers with circulating fibrocytes of IPF.

**Results:**

Using a gating strategy for both circulating and lung fibrocytes, which excludes potentially contaminating cell populations (e.g. neutrophils and different leukocyte subsets), we show that patients with IPF have increased proportions of fibrocytes, not only in the circulation, but also in explanted end-stage IPF lungs. These lung fibrocytes have increased surface expression of HLA-DR, increased intracellular collagen-1 expression, and also altered forward and side scatter characteristics compared with their circulating counterparts.

**Conclusions:**

These findings demonstrate that lung fibrocytes in IPF patients can be quantified and characterized by flow cytometry. Lung fibrocytes have different characteristics than circulating fibrocytes and represent an intermediate cell population between circulating fibrocytes and lung fibroblast. Therefore, more insight in their phenotype might lead to specific therapeutic targeting in fibrotic lung diseases.

**Electronic supplementary material:**

The online version of this article (10.1186/s12931-018-0798-8) contains supplementary material, which is available to authorized users.

## Background

Fibrocytes are thought to be the precursors of fibroblasts and were first described in an experimental skin wound model in mice as bone marrow-derived cells producing extracellular matrix proteins aiding wound healing [1]. Fibrocytes are derived from a common monocyte lineage [2] and upon tissue injury, they migrate to the affected organ in response to chemotactic factors, where they differentiate into fibroblast-like cells. Next to differentiation into (myo) fibrobalsts, fibrocytes are thought to display several paracrine functions, including fibroblast activation, alternative macrophage-dependent and -independent inflammatory processes, which all could lead to tissue remodeling and fibrosis [3–7]. The contribution of fibrocytes to the pathogenesis of fibrotic diseases and their potential use as a biomarker in fibrotic lung diseases and pulmonary hypertension (PH) has investigated, as they correlate to disease progression and survival [8–11].

However, a uniform (gating) strategy to identify fibrocytes is lacking. It is generally accepted that the minimally needed markers are CD45 (hematopoietic marker) and intracellular collagen-1 [12–14]. Discrepancies in opinion how to further accurately characterize fibrocytes may originate from two possible problems. First, it is unclear whether other extracellular markers are really needed, and if so, which ones would then be the most optimal. Most often CD34 (hemopoietic stem cell marker) and CXCR4 (C-X-C chemokine receptor 4), which are expressed on almost all circulating fibrocytes, are used. Secondly, there is no consensus whether circulating fibrocytes are cells with high side scatter (SSC) characteristics, a measure of cell granularity or internal complexity. Most studies have shown that CD45^+^/collagen-1^+^ fibrocytes represent a heterogeneous cell population primarily found in the polymorphonuclear (PMN) cell fraction with high SSC [8, 9, 13–15]. In contrast, others have demonstrated that circulating fibrocytes share side scatter characteristics comparable with blood mononuclear cell fraction based on cell sorting experiments [16, 17]. As a consequence, it is unclear whether differences in SSC represent different subpopulations, different stages of development or whether this reflect methodological issues.

Lung fibrocytes may hold promise in a better understanding of fibrocyte biology, as they have become fully differentiated effector cells and their paracrine and inflammatory function have taken shape.

Increased CD45^+^/collagen-1^+^ fibrocytes have been found in bronchoalveolar lavage (BAL) of IPF patients [18]. However, whether lung fibrocytes can be detected in IPF lung tissue homogenates using flow cytometry is currently unknown.

In our study, we propose a gating strategy and phenotypic staining for a more specific selection of circulating and lung fibrocytes and compare proportions and phenotypical characteristics of lung fibrocytes in IPF lung tissue with circulating fibrocytes in patients with IPF. Reliably identification of circulating and lung fibrocytes could be of great value of developing new therapies that target circulating and lung fibrocytes in IPF.

## Materials and methods

### Study design and subjects

Human lung tissue was collected from patients with end-stage IPF undergoing lung transplantation. As a control, lung tissue was obtained from long volume reduction procedures during lung transplantation upon size mismatch of oversized donor lungs or residual material obtained during lung surgery for pulmonary tumors. Healthy residual tissue was obtained at least > 3 cm from the tumor and only patients with a normal pulmonary function test (PFT) or mild airflow obstruction (Gold 1) were selected. All patient and healthy subject characteristics are shown in Additional file 1.

The Medical Ethical Committee of the Erasmus MC Rotterdam approved this study (*METC 2012–512*). Informed consent was obtained from every participant and healthy control (HC) before collection of blood samples. The diagnosis of pulmonary hypertension or pulmonary fibrosis was conform the current diagnostic guidelines of the ATS/ERS [19–21].

### Blood processing

Blood samples were collected in EDTA tubes (BD Vacutainer K2E). Peripheral blood mononuclear cells (PBMC) and total white blood cells were obtained according to standard protocols. In short, PBMC were obtained using the Ficoll separation technique and whole white blood cells with the simple Pasteur pipette tube technique after spinning samples at 1000 x g as previously described [22]. Red blood cells were lysed using osmotic lysis buffer (8.3% NH_4_CL, 1% KHCO_3_, and 0.04% NA_2_EDTA in Milli-Q). Upon isolation, PBMC and total white blood cells were resuspended in 0.1% BSA + 2 mM EDTA in PBS and immediately processed for flow cytometry and fluorescence-activated cell sorting (FACS). Isolated PBMC or total white blood cells, not used for direct flow cytometry or FACS, were aliquoted and cryopreserved in complete RPMI (RPMI medium 1640 + glutaMax, Life Technologies) with 10% DMSO (Sigma), 40% Fetal calf Serum (FCS) and stored at − 196 °C until thawing.

### Lung tissue processing

Fresh lung tissue was stored in cold PBS and processed within 24 h following lung transplantation or resection. Lung tissue was processed for isolation of single cell suspensions described in section *“preparation of single cell suspensions”*. Furthermore, peripheral lung tissue was frozen and stored at − 80 °C until further use.

### Preparation of single cell suspensions

Lung resection specimens were rinsed with PBS to remove residual blood. After mincing the lung, specimens were enzymatically digested in digestion medium (20 ml HBSS (Life Technologies, 14,170–088) with 10μg Liberase (Roche, Liberase™, research grade) and 40 Units of DNA-se (Roche, DNase I recombinant, RNase-free)) for 30 min in a humidified incubator at 37 °C while gently shaking the samples. The remaining cell debris was removed by passing the cells through a 100 μm-diameter disposable cell mesh filter. The cells were washed in RPMI with 5% FCS and centrifuged for 10 min at a speed of 400×g. Samples were subjected to RBC lysis, washed and counted. Finally, samples were aliquoted and cryopreserved in complete RPMI with 10% DMSO (Sigma), and 40% Fetal Calf Serum (FCS) and stored at − 196 °C.

### Human fibrocyte and human fibroblast culture

Human fibrocytes were cultured from peripheral blood as previously described [7], with some modifications. Briefly, following Ficoll density centrifugation, 2 × 10^5 PBMCs were plated into culture-slides (sigma, C7182, 0.8 cm^2^/well) in complete culture medium (Dulbecco’s modified Eagle’s medium) (DMEM) supplemented with 20% fetal calf serum, 2 mM L-glutamine, 100 U/mL of penicillin, 100 mg/mL of streptomycin) (Life Technologies, Grand Island, NY) at 37 °C and 5% CO2. After 3 days, non-adherent cells were aspirated and discarded and fresh medium was applied. Following 10–14 days of culture, slides were washed three times with ice-cold PBS and the chambers were removed from the glass. Normal human lung fibroblasts (NHLF) were also cultured on culture slides (50,000 NHLF/well) in complete culture medium. NHLF were donated from the Laboratory of the University of Virginia (School of Medicine, Charlottesville, VA, USA.) After 2–3 days non-adherent NHLF were aspirated and discarded. Subsequently, the cultured NHLF were washed with ice-cold PBS, dried and and processed similarly to the cultured human fibrocytes.

### Flow cytometry and FACS staining

Freshly isolated PBMCs and total white blood cells were stained for extra- and intracellular markers using the following antibodies: CD45-V450 (HI30), CD56-Af488 (B159 RUO), CD15-PE (HI98), CD16-PerCP-Cy5.5 (3G8), HLA-DR-BV711 (G46–6), CXCR4 (CD184)-Pe-Cy7 (12G-5) (BD Biosciences), strep-APC-eF780, CD3-FITC (UCHT1), CD19-FITC (HIB19) (eBiosciences), Collagen-1-Biotin conjugated Bio (Rockland, 600–406-103), CD14-PE-Texas Red (Tuk4) (Invitrogen). To control for non-specific labeling Rabbit IgG-Biotin conjugated (Rockland, 011–0602) was used. For the cell sorting experiments, the same extra-cellular antibodies were used, except for HLA-DR: HLA-DR-APC (G46–6) (BD Biosciences).

Since macrophages in lung single cell suspensions have high auto fluorescence, we did not use fluorochromes FITC and Alexa Fluor 488 in the staining and used the following antibodies: CD3-BV711 (UCHT1), CD19-BV786 (J25C1), CD56-BV605 (NCAM16.2) (BD Biosciences). Fixable Viability Dye eFluor 506 (eBiosciences) was applied as a live-dead marker for flow cytometry experiments and 4′,6-diamidino-2-phenylindole (DAPI) (Invitrogen, Molecular Probes) was used as live-dead marker for the cell sorting experiments. In short, cells were incubated in FACS buffer (PBS, 0.25% BSA, 0.5 mM EDTA, 0.05% NaN3 sodium azide) with fluorescent antibodies for 30 min at 4 °C using methods recommended by the manufacturers. Of note, extra-cellular CXCR4-Pe-Cy7 staining was performed separately in MACS buffer (0.5% BSA + 2 mM EDTA in PBS). After fixation and permeabilization (BD Cytofix/Cytoperm kit, 554,714), cells were incubated with the biotinylated Collagen-1 antibody or Isotype control in permeabilization buffer for 30 min at 4 °C. Biotinylated antibodies were visualized with streptavidin-APC-eF780. Cells were measured on a either a LSRII or a FACS Aria™ IIu Flow cytometer (both BD Biosciences). We analyzed a minimum of 200,000 alive cells for blood samples and 100,000 alive cells for the lung tissue samples for cytometric analysis. Data was analyzed by FACS Flow-Jo software.

### Cytospin

Sorted cells were washed in PBS. Cytospins were made using a cytocentrifuge and 50,000 cells were added per spot. Slides were air-dried and stored at − 80 °C in a watertight box until further use. Immunocytochemistry was performed within 4 weeks after storing at − 80 °C.

### Immunocytochemistry

Cytospin-slides were fixed in 100% acetone at room temperature for 15 min. For the collagen-1 staining, slides were pre-incubated with 10% normal goat serum (Sigma, G9023) in block buffer (1% Blocking Reagent, Roche, in PBS according to the manufacturer’s protocol) for 30 min. After rinsing with PBS, slides were incubated for 60 min with mouse anti-human collagen-1 (Abcam, ab6308, 1:2000) or Isotype control in block buffer. As second (goat anti-mouse antibody biotin-labeled) and third antibody (streptavidin, alkaline phosphatase (AP) conjugate) we used the Link-Label kit from Biogenex (link: HK-325-UM, label HK321-UK) according to the manufacturer’s protocol. To detect the collagen-1 positive cells we used New Fuchsin Alkaline Phosphatase Substrate Solution (0.01% New Fuchsin, 0.02% Sodium Nitrite, 0.03% Naphthol AS-BI Phosphate, 1 mM Levamisole, in 0.2 M Tris-HCl, pH 8.5). Cells were counterstained with hematoxylin (Sigma, Gill No. 3), dried and mounted in Vecta Mount (Vector, Burlingame, CA, USA). For CD15 detection we used the same protocol, but with different antibodies; slides were pre-incubated with normal rabbit serum (Sigma, R9133) and subsequently stained with mouse anti-human CD15-FITC (BD Biosciences, HI98) or isotype control and rat anti-FITC AP conjugate (Sigma, A4843).

### Statistics

Statistical analysis was performed using IBM SPSS Statistics 21 and GraphPad Prism 6 software. When evaluating differences in continuous variables between multiple independent groups, the Kruskal-Wallis test was used. For calculating the level of significance of differences between groups we used the Mann-Whitney U test. Correlation coefficients were calculated using Spearman’s rank method. *P* values < 0.05 were considered significant. Flow cytometry data is either represented as percentage population or as mean fluorescence intensity (MFI).

## Results

### Circulating CD45^+^/Col-1^+^ fibrocytes may be contaminated with polymorphonuclear leukocytes

**Fig. 1:**
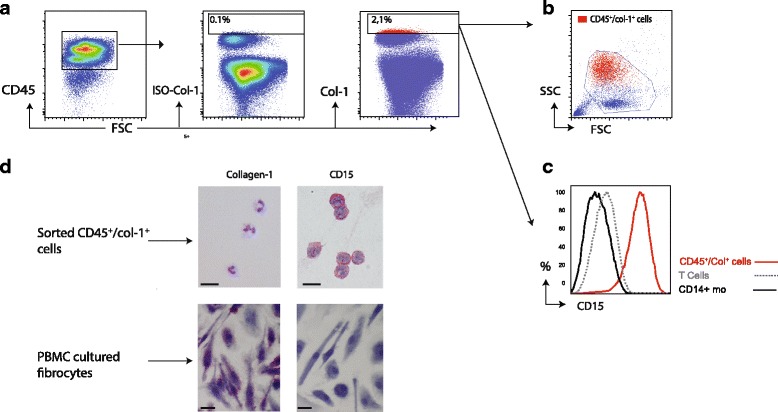
Circulating CD45^+^/Col-1^+^ fibrocytes are contaminated with polymorphonuclear leukocytes. **a** Representative gating strategy for identification of circulating CD45^+^/Collagen-1^+^ fibrocytes from PBMCs. Isotype control for collagen-1 (Col-1) was used to set the gate for Col-1+ cells within alive CD45^+^ cells. Red cells are CD45^+^Col-1^+^. **b** FSC and SSC characteristics of CD45^+^/Col-1^+^ cells (in red) compared to all alive cells (blue) showing that most CD45^+^/Col-1^+^ cells are found in the polymorphonuclear leukocytes fraction. **c** Histogram overlay showing surface expression of CD15 assessed by flow cytometry on CD45^+^/Col-1^+^ cells (red), CD14+ monocytes (black) and T cells (gray). **d** CD45^+^/Col-1^+^ cell enriched fraction and PBMC cultured fibrocytes were analyzed with immunocytochemistry (ICC) for CD15 and collagen-1 expression. Magnification for all ICC figures was 200× and sections were counterstained with hematoxylin. This is representative of 7 experiments CD14+ Mo = CD14+ monocytes, PBMC = peripheral blood mononuclear cells, FSC = forward scatter, SSC = side scatter.

Since discrepancies have been reported about fibrocytes concerning their granularity and/or internal complexity, we first evaluated SSC characteristics of fibrocytes identified based on CD45 and collagen-1 (Col-1) expression. Fibrocytes were detected using the gating strategy shown in Fig. 1a. Col-1 expression was based on the control isotype staining. Circulating CD45^+^/Col-1^+^ fibrocytes represented a heterogeneous cell population based on SSC and have predominantly a high SCC (Fig. 1b) Because SSC-high cells contain polymorphonuclear cells, such as neutrophils, we examined the adhesion molecule CD15, which is expressed on circulating neutrophils [23]. The CD45^+^/Col1^+^ cells showed a high extracellular expression level of CD15 (Fig. 1c). To investigate whether this population could be contaminated with neutrophils, we isolated circulating CD45^+^/Col-1^+^ cells based on extracellular markers (sort strategy shown in Additional file 2) and analyzed these cells with immunocytochemistry (Fig. 1d). Almost all cells (98,6, 95% CI 97,9–99,2) in the flowcymetric enriched CD45^+^/Col-1^+^ population were negative for collagen-1 and positive for CD15 with immunocytochemistry, whereas cultured fibrocytes (Fig. 1d) and fibroblasts (Additional file 3) were positively stained for collagen-1 and negative for CD15. Additionally, all cells in the enriched CD45^+^/Col-1^+^ group had a multi-lobulated shaped nucleus. We also found a significant correlation between circulating CD45^+^/Col-1^+^ cells and neutrophils (R = 0.39, p = 0.006) (Additional file 4).

In conclusion, our data show that PMN-leukocytes and especially neutrophils contaminate fibrocyte identification when using only CD45 and collagen-1 as identification markers. Consequently percentages of fibrocytes in the circulation are most likely lower than previously reported.

### Identification and characterization of lung fibrocytes in IPF lungs

As neutrophils hamper the identification of fibrocytes in peripheral blood, we developed a strategy to selectively identify fibrocytes. Since circulating fibrocytes are a putative source for fibroblastic foci, a hallmark of IPF, we used IPF lungs to test our gating strategy. Lung fibrocytes in IPF lungs initially maintain CD45 expression and their presence has been previously confirmed with immunofluorescence [11, 24]. We obtained single-cell suspensions of explanted IPF lungs (*n* = 3). As a control we used healthy lung tissue from volume reduction procedures during lung transplantation or residual material of patients who underwent a lobectomy for lung cancer, hereafter called control lungs (*n* = 4).

**Fig. 2:**
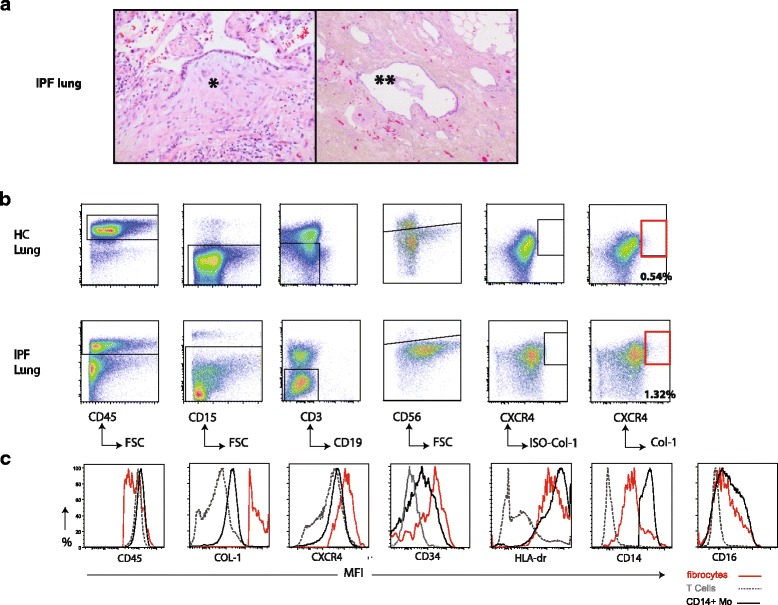
Identification and characterization of lung fibrocytes in IPF lungs. a Representative histological picture of an explanted end-stage IPF lung showing (left): a usual interstitial pneumonia (UIP) pattern with (*) **a** fibroblast focus with overlying reactive epithelium and (right): (**) area of completely fibrotic remodeled lung tissue with cyst formation and bronchiolisation, amounting to honeycombing. Magnification 20× (left) and 5× (right). **b** Representative gating strategy for lung fibrocytes (CD45^+^CD15^−^CD3^−^CD19^−^CD56^−^CXCR4^+^Col-1^+^-cells) in explanted IPF lungs (*n* = 3 bottom) and control lungs (HC, top) (*n* = 4). Single cell suspensions were thawed and alive viable cells were further analyzed. Lung fibrocytes are present in the CD45^+^ cell population. Contaminating and unwanted CD15^+^ neutrophils, CD3^+^ T cells, CD19^+^ B cells and CD56^+^ NK-cells were sequentially excluded. Isotype control for Col-1 was used to set the gate for Col-1+ cells. **c** Representative histogram overlay showing expression levels depicted as MFI of indicated markers assessed with flow cytometry of lung fibrocytes (red), T cells (gray) and CD14+ monocytes (black). CD14+ Mo = CD14+ monocytes, MFI = mean fluorescence intensity

All explanted lungs of IPF patients used in this study were reviewed by a pathologist and fulfilled the criteria for an usual interstitial pneumonia (UIP) pattern (Fig. 2a). We next evaluated whether we could selectively identify fibrocytes after exclusion of neutrophils, T cells, NK cells, and B cells. Using this strategy, we could distinguish a fibrocyte population expressing CD45, CXCR4 and intracellular collagen-1 in both IPF lungs and control lungs (Fig. 2b).

Fibrocytes express CD45, CXCR4 and CD34 and intracellular collagen-1 and are generally believed to mature from a subpopulation of CD14+ mononuclear cells [25]. Figure 2c shows the expression level of these markers and HLA-DR, CD14, CD16 on lung fibrocytes (CD45^+^CD3^−^CD19^−^CD56^−^CD15^−^CXCR4^+^Col-1^+^). CD45 expression on lung fibrocytes was slightly lower than on T-cells and CD14+ monocytes. As expected, collagen-1 and CXCR4 were clearly expressed by lung fibrocytes compared to CD14+ monocytes and T cells. CD34, a commonly used progenitor cell marker, is expressed on fibrocytes when they have entered the lungs. HLA-DR expression on lung tissue-resident fibrocytes is similar to CD14+ monocytes and higher compared to T cells of the same donor. Tissue-resident fibrocytes showed an intermediate expression of CD14 (expression level between CD14+ monocytes and T cells, which are CD14 negative) and relatively low expression of CD16.

In conclusion, lung fibrocytes can be detected in lung single cell suspension after exclusion of neutrophils, T cells, NK cells and B cells. Lung fibrocytes express known fibrocyte surface markers such as CD34, CD45, and CXCR4, and intracellular marker Col-1, suggesting that they have just entered the lung tissue and have not differentiated into myofibroblasts yet. The high expression by HLA-DR of lung fibrocytes suggests that they have a potential role in antigen presentation.

### Detailed identification of circulating fibrocytes

Having shown that fibrocytes can be reliably detected in lung single-cell suspensions after exclusion of neutrophils and lymphocytes, we hypothesized that this would also be applicable for the detection of circulating fibrocytes. Next to IPF patients, we investigated fibrocytes in pulmonary hypertension (PH) patients, as elevated number in the periphery have been observed before [10]. We examined fresh PBMC of IPF patients (*n* = 5), PH patients (*n* = 4) and healthy controls (HC)(n = 4). Patient characteristics are detailed in Additional file 1: Table S1.

**Fig. 3:**
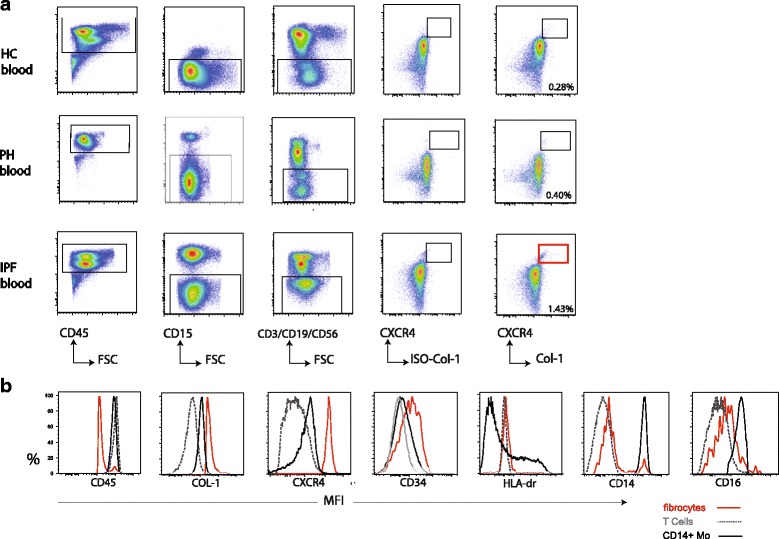
Characterization of circulating fibrocytes in IPF and PH patients. **a** Representative gating strategy for the detection of circulating fibrocytes (CD45^+^CD15^−^Lin^−^CXCR4^+^Col-1^+^-cells) in freshly analyzed PBMCs of heathy controls (*n* = 5), PH patients (n = 5) and IPF patients (n = 4). The lineage mix includes markers CD3, CD19 and CD56 to exclude T-cells, B-cells and NK-cells. **b** Representative histogram overlay showing expression levels depicted as MFI of indicated markers assessed with flow cytometry of circulating fibrocytes (red), T cells (gray) and CD14+ monocytes (black)

A representative dot-plot of the gating strategy to identify circulating fibrocytes (CD45^+^lin^-^CD15^-^CXCR4^+^Col-1^+^-cells) is shown in Fig. 3a. (Lineage mix contains: CD3, CD19, and CD56) A well-defined population of circulating fibrocytes was identified in PBMC fractions of HC, IPF and PH patients.

Next, we investigated the expression profile of circulating fibrocytes. Circulating fibrocytes expressed high levels of collagen-1, CXCR4 and CD34 and lower levels of CD45 compared with CD14+ classical monocytes and T cells (Fig. 3b). HLA-DR expression on circulating fibrocytes was comparable to HLA-DR expression on T cells, whereas CD14+ classical monocytes contained cells with high, low, and intermediate HLA-DR expression. Interestingly, the majority of circulating fibrocytes have a low expression of CD14 and only a small proportion (11,3 95% CI 9,7–13,0%) expressed CD14 comparable to classical monocytes. CD16 expression on circulating fibrocytes was lower compared to classical monocytes and slightly higher than T cells of the same donor.

In conclusion, these data show that circulating fibrocytes can be detected after exclusion of neutrophils, T, B and NK cells, and express markers in common with lung fibrocytes. HLA-DR and CD16 expression are low on circulating fibrocytes and only a small fraction expresses high levels of CD14.

### Quantification of fibrocyte numbers is independent of leukocyte isolation strategy used

**Fig. 4:**
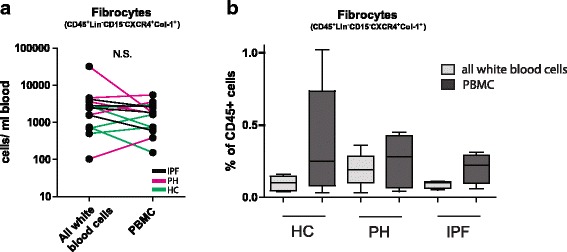
The total number of circulating fibrocytes is unaffected by the leukocyte isolation strategy used. **a** Total number of circulating fibrocytes (defined as CD45^+^lin^−^CD15^−^CXCR4^+^Col-1^+^ cells) per ml blood using two common leukocyte isolation techniques, e.g. the simple Pasteur pipette tube technique to isolate all white blood cells and Ficoll separation technique to isolate PBMCs. For this experiment we analyzed paired total white blood cells and PBMCs on the same day as blood withdrawal of 9 patients (4 IPF patients (black) and 5 PH patients (purple)) and 5 healthy controls (green). **b** Percentage of fibrocytes (of CD45^+^ cells) in the same patients after isolating all white blood cells (gray) and PBMCs (black). Data are depicted as median and interquartile

Since the presence of neutrophils hampers the detection of fibrocytes and to confirm the specificity of our staining, we compared two common leukocyte isolation techniques. We compared the Ficoll separation technique to isolate PBMCs to remove PMN-leukocytes, and the simple Pasteur pipette tube technique to isolated all white blood cells. We analyzed paired total white blood cells and PBMCs on the same day as blood withdrawal of 9 patients (4 IPF patients and 5 PH patients) and 5 healthy controls. The absolute number of circulating fibrocytes (CD45 + Lin-CD15-CXCR4 + Col-1+) per milliliter (ml) blood was not different between the two leukocyte isolation techniques (2,5 × 10^3^ (95% CI -0,4 × 10^3^–9,0 × 10^3^)(Pasteur pipette technique) versus 1,8 × 10^3^ (95% CI 1,1 × 10^3^–1,7 × 10^3^)(Ficoll separation technique)) (Fig. 4a). As expected, the proportions of circulating fibrocytes from CD45+ cells was relatively higher in PBMC samples than in whole blood samples, because the Ficoll technique eliminated most PMN-leukocytes (Fig. 4b). The lack of difference in fibrocytes percentages in this figure between groups of patients and controls is probably the result of the low number of patients used for this experiment.

In conclusion, our strategy to identify fibrocytes is reliable, irrespective of which leukocyte isolation technique is used, for the quantification of absolute numbers of circulating fibrocytes.

### Comparison of circulating fibrocytes with lung fibrocytes

Upon tissue injury, fibrocytes migrate to target organs and mature in (myo) fibroblasts and participate in tissue remodelling and fibrosis. It is unclear if circulating fibrocytes become more activated, or modulate surface markers (e.g. CD45, CD34 or CXCR4), or upregulate intracellular collagen-1 when entering the lung. Therefore we simultaneously compared the expression levels of these markers between circulating and tissue-resident fibrocytes.

**Fig. 5:**
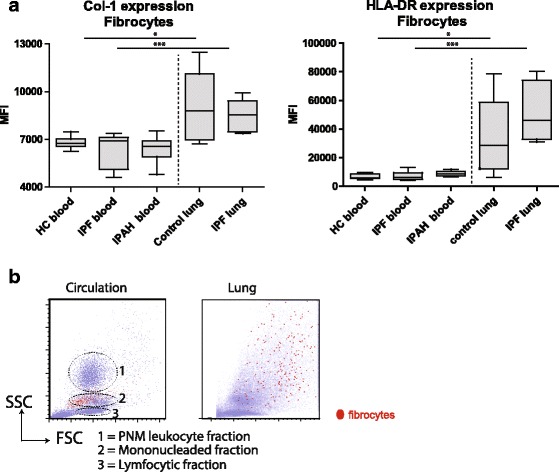
Comparison of circulating fibrocytes with lung fibrocytes. **a** Intracellular Collagen-1 and surface HLA-DR expression analyzed with flow cytometry and depicted as MFI. For this experiment, we used frozen PBMC of 10 HC, 10 IPF patients and 10 IPAH patients and frozen single cells suspensions of 5 control lungs and 5 explanted IPF lungs. **b** representative dot-plot of the FCS and SSC characteristics assessed with flow cytometry of circulating and lung fibrocytes (red dots) in the circulation (left panel) and explanted IPF-lung (right panel). Nonparametric two-tailed Mann-Whitney test was used. Data are depicted as median and interquartile. * *P* < 0.05 *** *P* < 0.001IPAH = idiopathic pulmonary arterial hypertension, PMN = polymorphonuclear.

Collagen-1 expression in fibrocytes did not differ between IPF/(IPAH) patients and (healthy) controls in either lung cell suspensions or PBMCs (Fig. 5a, left). The collagen-1 expression in lung fibrocytes was significantly higher compared with circulating fibrocytes in patients with IPF, IPAH and controls.

Expression of HLA-DR was similar between IPF/IPAH patients and controls (Fig. 5a, right). Tissue-resident fibrocytes showed a significantly increased expression of HLA-DR compared to circulating fibrocytes. CD34 and CXCR4 expression between circulating and tissue-resident fibrocytes did not differ (data not shown).

To examine the size and complexity of circulating and lung fibrocytes, we examined SSC and FSC of the fibrocytes selected as shown in Figs. 2b and 3a. Circulating fibrocytes had FSC and SSC characteristics comparable to monocytes. In the lung, both FSC and SSC of lung fibrocytes were increased compared to circulating fibrocytes (Fig. 5b). The variation in FSC and SSC characteristics of lung fibrocytes could be the result of differences in granularity, activation status and collagen content.

In conclusion, these data show that lung tissue-resident fibrocytes have increased expression levels of HLA-DR and collagen-1 and also gain size and internal complexity compared with their circulating counterpart. No differences were observed within a compartment between controls or patients with IPF or IPAH, which may suggest an important role of the local environment in lungs on fibrocyte development.

### Increased proportions of fibrocytes in the lungs and circulation of patients with IPF

Having shown that our gating strategy to detect fibrocytes is reliable and specific, we wanted to apply this technique in a larger, clinically relevant, cohort of PH and IPF patients, in which increased percentages of circulating fibrocytes have been described before [8, 10, 12]. To minimize variability in fibrocytes due to heterogeneity in etiology of PH, only patients with idiopathic arterial pulmonary hypertension (IPAH) were examined. We thus determined fibrocyte percentages in PBMCs of patients with IPF (*n* = 14), IPAH (*n* = 10), and HC (n = 10).

**Fig. 6:**
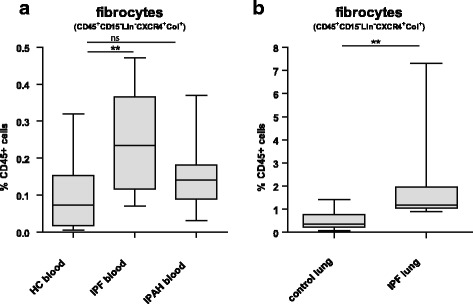
Circulating and Lung fibrocyte numbers in patients with IPF and idiopathic pulmonary hypertension (IPAH). **a** Percentage of circulating fibrocytes of CD45+ cells in frozen PBMC of HC, patients with IPF and patients with IPAH. **b** Percentage of lung fibrocytes of CD45+ cells in frozen single cell suspensions of control lungs (*n* = 9) or end-stage explanted IPF lungs (*n* = 8). Nonparametric two-tailed Mann-Whitney test was used. Data are depicted as median and interquartile. ** *P* < 0.01

Circulating fibrocytes are significantly increased in patients with IPF compared with control samples (*p* < 0.01) (Fig. 6a and Additional file 5). The average percentage of circulating fibrocytes in IPF patients was 0.25% (95% CI 0.17–0.33) of all CD45+ cells, compared with 0.10% in HC (95% CI 0.03–0.17). The percentage of circulating fibrocytes in IPAH patient was 0.18% (95% CI 0.08–0.22) and not increased compared with HC (*p* = 0.14).

In line with previous literature, we have shown that circulating fibrocytes are increased in patients with IPF. We next evaluated whether lung fibrocytes would also be increased in lungs of patients with IPF (*n* = 8) and compared them to control lungs (*n* = 9) (Fig. 6b). Clinical characteristics of these patients are shown in Additional file 1.

Indeed, the percentage of lung fibrocytes in IPF lungs was 2,6% (95% CI -0.8-5.9) of all CD45+ cells, which was increased (*p* = 0.002) compared with control lungs (0.7%, (95% CI 0.02–1,3%)).

Collectively, these data show that patients with IPF have increased proportions of fibrocytes, not only in the circulation, but also in the lungs at end-stage disease.

## Discussion

To our knowledge, this is the first time that lung fibrocytes in IPF lung tissue homogenates is assessed by flow cytometry. We have shown that lung fibrocytes are elevated in IPF lungs and express higher levels of HLA-DR and collagen-1 and gain size and internal complexity compared with circulating fibrocytes. We have used a reliable gating strategy, which excludes disruptive cell populations, and especially granulocytes, for a specific identification of circulating and lung fibrocytes.

Identifying circulating fibrocytes using flow cytometry is challenging since no uniform gating strategy exists, leading to a high variability in circulating fibrocyte numbers in various reports. Circulating fibrocytes are bone marrow-derived monocyte-like cells capable of producing components of the extra-cellular matrix, and therefore at least CD45 and intracellular Collagen-1 should be used to identify circulating fibrocytes. In addition, CD34 and a variety of chemokine receptors have been used to identify subtypes of circulating fibrocytes, most notably CXCR4, CCR7, and CCR2. In the present study, we have shown that CXCR4 is equally expressed on lung and circulating fibrocytes irrespective of underlying disease. Most circulating fibrocytes also express CD34 [5, 16, 26]. We have shown that lung fibrocytes also expresses CD34. CD34 binds to L-selectin and in L-selectin KO-mice fibrosis induction is hampered in the bleomycin exposure model of lung fibrosis [27]. This suggest that CD34 might be needed for circulating fibrocytes to enter the lung tissue via L-selectin expressed on activated endothelial cells. It is unclear if or at what point CD34 may be downregulated in vivo*, a*s Andersson-Sjoland and colleagues have shown by demonstrating that mature alfa-SMA positive fibrocytes in IPF lungs still express CD34 [24]. In vitro*,* CCR7 expression correlates with increased migration of circulating fibrocytes and TGF-β production [28]. The CCR2-CCL2 axis promotes fibrocyte recruitment and induces their differentiation into (myo) fibroblasts [29, 30]. Therefore, it is believed that these chemokines receptor-bearing fibrocytes may represent a more activated subtype, however expression levels of these markers varies between different diseases and the majority of fibrocytes do not express CCR2 and CCR7 [6, 7, 28, 31, 32]. The gating strategy described here could be used for reliable determination of these homing receptors and other markers of interest.

Our study has shown that the circulating fibrocyte pool is contaminated with granulocytes when employing a gating strategy based on CD45 and collagen-1 alone. Contamination of granulocytes is most likely the result of non-specific binding of collagen-1 antibody to granulocytes. Neutrophils, the most prevalent granulocytes, express a variety of collagen receptors and play an important role in collagen breakdown. However, to our knowledge there are no reports of collagen-containing neutrophils, suggesting that antibody binding is non-specific [33]. It is well known that Fc Receptors on granulocytes can cause nonspecific, false-positive antibody staining, even in the presence of specific blocking reagents [34]. Presumably, in very ill patients granulocytes could express more Fc-receptors compared with healthy subjects [35, 36]. Additionally, we observed that circulating fibrocytes had similar FSC and SSC characteristics compared to monocytes. Therefore, we strongly advocate the use of markers that exclude granulocytes in current gating protocols to identify fibrocytes.

In line with previous reports [8, 12], we found increased percentages of circulating fibrocytes in patients with IPF, but not in patients with IPAH. Yeager and colleagues described increased percentages of circulating fibrocytes in children with idiopathic or hereditable PH [10]. It is conceivable that in our relative small sample size, the extent of tissue remodeling or fibrosis in IPAH patients is relatively too low to pick up differences in circulating fibrocytes. Therefore, a larger cohort of patients is probably needed to investigate if circulating fibrocytes are also elevated in adults with IPAH.

The proportions of circulating fibrocytes we describe after eliminating disruptive cell populations is much lower than previously reported. Like macrophages, fibrocytes are monocyte-derived and their development into an effector cell is primarily established outside the circulation and depends on local tissue environment [37]. It is though that the monocyte-fibrocyte pathway may be similar and that the majority of fibrocytes is present in target organs. Indeed, we have shown that tissue-resident fibrocytes can be identified in IPF lungs using flow cytometry and that they make up a greater proportion of the CD45^+^ cell compartment compared with their circulating counterpart. These lung fibrocytes express high levels of collagen-1 compared to the circulating fibrocytes, which makes the collagen-1 staining more robust. HLA-DR expression is also upregulated in lung fibrocytes compared to circulating fibrocytes. As HLA-DR molecules can provoke or suppress T-(helper)-cell responses and are upregulated in response to signaling, the increased expression may point toward antigen-presentation or cross talk with local T cells in vivo. It has also been shown that cultured fibrocytes express all the necessary costimulatory molecules for antigen-presentation, that they are potent stimulators of naive T-cells and induce a Th2 cytokine response in vitro [38, 39]*.*

Collectively, this gating strategy holds great potential in investigating lung fibrocytes in more detail. This method could replace experiments on artificially cultured fibrocytes and allow for direct investigation of the role of lung fibrocytes in tissue remodeling and fibrosis in its target organ.

## Conclusion

Using a gating strategy that excludes possible contaminating cell populations, we show that lung fibrocytes in IPF lungs can be assessed by flow cytometry and that their phenotype differs from circulating fibrocytes. This new approach could be interesting for scientists investigating the fascinating role of fibrocytes in disease pathogenesis or their potential as a biomarker and therapeutic target.

## Additional files

Additional file 1: Characteristics of patients and the healthy subjects used for experiments. Data are mean ± standard deviation, unless indicated otherwise. ^a^ 1 patient with chronic thromboembolic PH and 4 patients with PH secondary to an auto-immune disease. ^b^ 6 patients with IPF, 3 patients with UIP pattern secondary to extrinsic allergic alveolitis, 2 patients with an non-specific interstitial pneumonia and 1 patient with anti-synthetase syndrome. ^c^ Mean FVC/FEV1 ratio = 0.71 (2 out of 9 had an obstructive pulmonary function test, both classified as GOLD A). ^d^ Assessed by right heart catheterization or suspected with echocardiography. IPF = idiopathic pulmonary fibrosis, IPAH = idiopathic pulmonary arterial hypertension, FVC = forced vital capacity, TLCO = diffusing capacity for carbon monoxide, PAP = pulmonary arterial pressure, RAP = right atrium pressure, Svo2 = mixed venous saturation, PDE5i = phosphodiesterase type 5 inhibitor, ERA = endothelin receptor antagonist, Pros-A = prostacyclin agonist (DOCX 22 kb)

Additional file 2: Sort strategy for CD45^+^/Col-1^+^ cells. (A) The conventional strategy with the CD45^+^/Col-1^+^ cells in red. (B) Position of the same fibrocytes when employing a gating strategy based on additional extracellular markers. The sort strategy is based on extracellular markers and with some modifications previously published [16] (of note: for our research question we did not exclude SSC^hi^ cells). CD45+ cells were analyzed for HLA-DR expression and lineage markers to exclude B-cells (CD19), NK cells (CD56) and T-cells (CD3). Lineage negative cells were plotted as CD14 versus CD16 to create a distinct group of cells enriched for CD45^+^/Col-1^+^ cells (red box). (PDF 75 kb)

Additional file 3: Complete overview collagen-1 and CD15 staining on CD45^+^/Col-1^+^ cells and controls. Immunocytochemical images of cultured fibroblasts, cultured fibrocytes, sorted classical monocytes and sorted CD45^+^/Col-1^+^ cells. Indicated cells were stained with Collagen-1 or isotype control (rabbit IgG) and CD15. As a control for CD15 we used a buffy coat, nicely showing positive granulocytes next to negative lymphocytes. Magnification for all images was 100×. (PDF 1282 kb)

Additional file 4: Correlation circulating CD45^+^/Col-1^+^ fibrocytes and granulocytes. For this experiment we analyzed paired total white blood cells (open dots) and PBMCs (black dots) on the same day as blood withdrawal of 9 patients (4 IPF patients and 5 PH patients). Correlation coefficients were calculated using Spearman’s rank method. (PDF 102 kb)

Additional file 5: Circulating fibrocyte numbers in patients with IPF and idiopathic pulmonary hypertension (IPAH). (A) Absolute numbers of circulating fibrocytes per ml blood in frozen PBMC of HC, patients with IPF and patients with IPAH. ** *P* < 0.01 (PDF 91 kb)

## **Abbreviations**

CCL: C-C motif ligand
CCR: C-C chemokine receptor
Col-1: collagen-1
CXCL: C-X-C motif ligand
CXCR: C-X-C chemokine receptor
FSC: forward scatter
HC: healthy control
IPAH: idiopathic pulmonary arterial hypertension
IPF: idiopathic pulmonary fibrosis
PBMC: peripheral blood mononuclear cell
PF: pulmonary fibrosis
PH: pulmonary hypertension
PNM: polymorphonuclear
SSC: side scatter
