# Supplementary material for: Fibrocytes are increased in lung and peripheral blood of patients with idiopathic pulmonary fibrosis
Source: Respir Res. 2018 May 10;19:90. doi: 10.1186/s12931-018-0798-8 (PMC5946532; doi:10.1186/s12931-018-0798-8)
Supplement: Supplementary file 1 — Characteristics of patients and the healthy subjects used for experiments. Data are mean ± standard deviation, unless indicated otherwise. a 1 patient with chronic thromboembolic PH and 4 patients with PH secondary to an auto-immune disease. b 6 patients with IPF, 3 patients with UIP pattern secondary to extrinsic allergic alveolitis, 2 patients with an non-specific interstitial pneumonia and 1 patient with anti-synthetase syndrome. c Mean FVC/FEV1 ratio = 0.71 (2 out of 9 had an obstructive pulmonary function test, both classified as GOLD A). d Assessed by right heart catheterization or suspected with echocardiography. IPF = idiopathic pulmonary fibrosis, IPAH = idiopathic pulmonary arterial hypertension, FVC = forced vital capacity, TLCO = diffusing capacity for carbon monoxide, PAP = pulmonary arterial pressure, RAP = right atrium pressure, Svo2 = mixed venous saturation, PDE5i = phosphodiesterase type 5 inhibitor, ERA = endothelin receptor antagonist, Pros-A = prostacyclin agonist (DOCX 22 kb) [file 12931_2018_798_MOESM1_ESM.docx]

|  |  | BLOOD |  |  |  | LUNG |  | Pt for SORT |  |
| --- | --- | --- | --- | --- | --- | --- | --- | --- | --- |
|  |  | HC  (n=15) | IPF  (n=14) | IPAH  (n=10) | PH (non IPAH)^a^  (n=5) | Control lung^b^  (n=9) | IPF lung  (n=8) | HC  (n=11) | PF^c^  (n=11) |
| General | \| age (years) \| \| --- \| \| male/female (n)(% male) \| \|  \| | 43±10  6/9 (40) | 69±7  11/3 (79) | 48±17  1/10 (10) | 51±10  2/3 (40) | 61±6  5/4 (56) | 63±2  4/7 (36) | 33±6  4/7 (36) | 69±8  3/8 (27) |
| Functional | \| FVC (% predicted \| \| --- \| \| TLCO (% predicted)  FEV1 (% predicted)  PH secondary to PF yes/no/nd (n)^d^  Mean PAP (mmHg)  RAP (mmHg)  Cardiac index (L/min/m2)  SvO2 (%)  NYHA-class 2/3 (n)   \|  \| \| --- \| \| |  | 66±16  37±14  1/0/9 | 55±14  12±6  2.1±0,9  62±7  3/7 | 44±11  8±1  2.9±0,8  67±7  2/3 | 105±17  80±21  105±15 | 50±11  35±8  56±12  3/4/1 |  | 68±18  35±8  3/6/2 |
| medication | Steroids  Pirfenidone  Nintedanib  No treatment  PDE5i  PDE5i + ERA  PDE5i +ERA + Pros-A (n) |  | 2  4  1  7  0  0  0 | 0  0  0  0  2  6  2 | 0  0  0  0  1  3  1 | 0  0  0  0  0  0  0 | 3  0  2  4  0  0  0 |  | 3  5  0  3  0  0  0 |
