# Supplementary figures and images for: Fibrocytes are increased in lung and peripheral blood of patients with idiopathic pulmonary fibrosis
Source: Respir Res. 2018 May 10;19:90. doi: 10.1186/s12931-018-0798-8 (PMC5946532; doi:10.1186/s12931-018-0798-8)

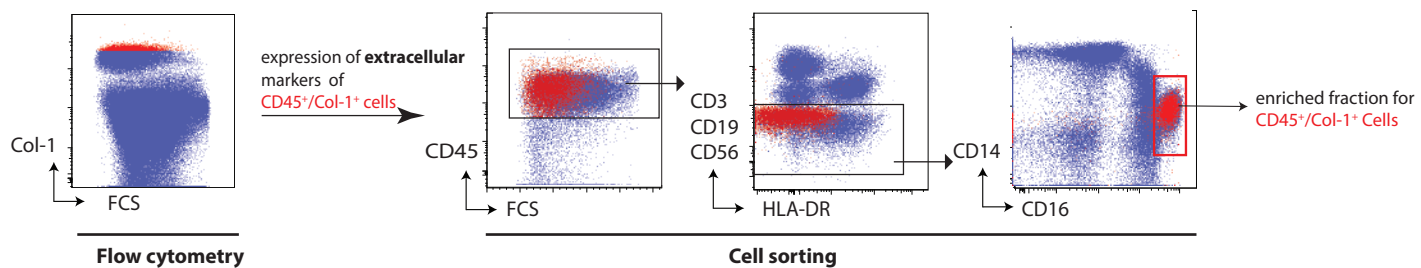

Supplement: Supplementary file 2 — Sort strategy for CD45+/Col-1+ cells. (A) The conventional strategy with the CD45+/Col-1+ cells in red. (B) Position of the same fibrocytes when employing a gating strategy based on additional extracellular markers. The sort strategy is based on extracellular markers and with some modifications previously published [16] (of note: for our research question we did not exclude SSChi cells). CD45+ cells were analyzed for HLA-DR expression and lineage markers to exclude B-cells (CD19), NK cells (CD56) and T-cells (CD3). Lineage negative cells were plotted as CD14 versus CD16 to create a distinct group of cells enriched for CD45+/Col-1+ cells (red box). (PDF 75 kb) [file 12931_2018_798_MOESM2_ESM.pdf]

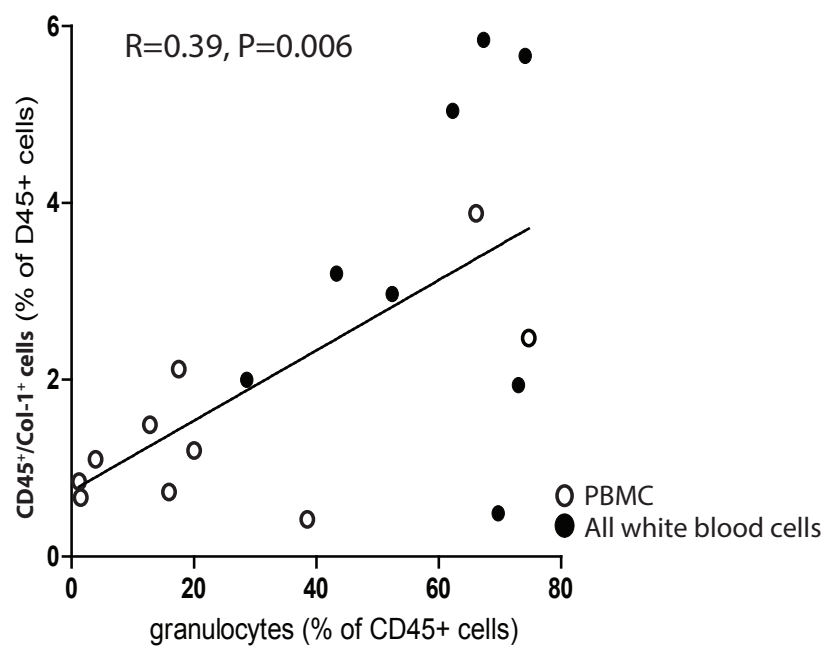

Supplement: Supplementary file 4 — Correlation circulating CD45+/Col-1+ fibrocytes and granulocytes. For this experiment we analyzed paired total white blood cells (open dots) and PBMCs (black dots) on the same day as blood withdrawal of 9 patients (4 IPF patients and 5 PH patients). Correlation coefficients were calculated using Spearman’s rank method. (PDF 102 kb) [file 12931_2018_798_MOESM4_ESM.pdf]

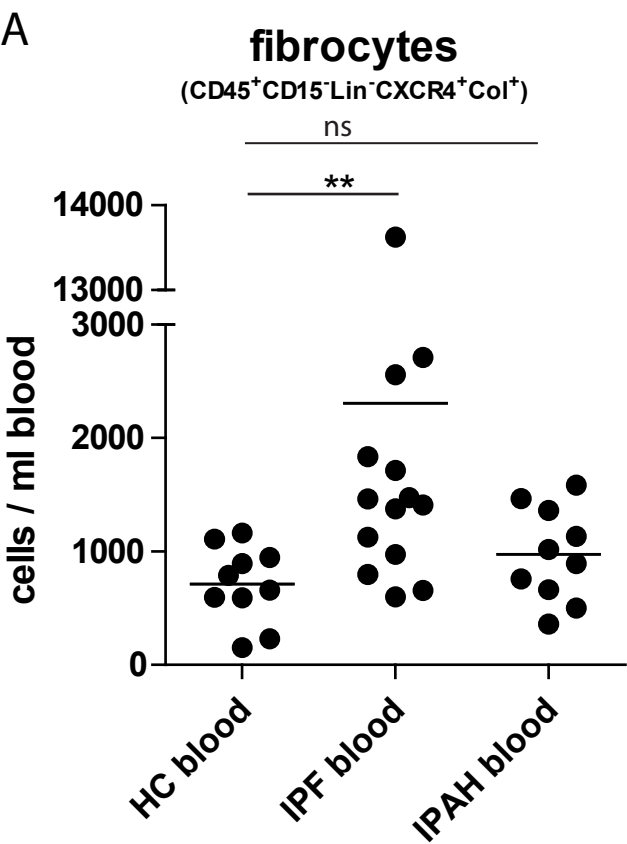

Supplement: Supplementary file 5 — Circulating fibrocyte numbers in patients with IPF and idiopathic pulmonary hypertension (IPAH). (A) Absolute numbers of circulating fibrocytes per ml blood in frozen PBMC of HC, patients with IPF and patients with IPAH. ** P < 0.01 (PDF 91 kb) [file 12931_2018_798_MOESM5_ESM.pdf]
