# Supplementary material for: Fibrocytes are increased in lung and peripheral blood of patients with idiopathic pulmonary fibrosis
Source: Respir Res. 2018 May 10;19:90. doi: 10.1186/s12931-018-0798-8 (PMC5946532; doi:10.1186/s12931-018-0798-8)
Supplement: Supplementary file 3 — Complete overview collagen-1 and CD15 staining on CD45+/Col-1+ cells and controls. Immunocytochemical images of cultured fibroblasts, cultured fibrocytes, sorted classical monocytes and sorted CD45+/Col-1+ cells. Indicated cells were stained with Collagen-1 or isotype control (rabbit IgG) and CD15. As a control for CD15 we used a buffy coat, nicely showing positive granulocytes next to negative lymphocytes. Magnification for all images was 100×. (PDF 1282 kb) [file 12931_2018_798_MOESM3_ESM.pdf]

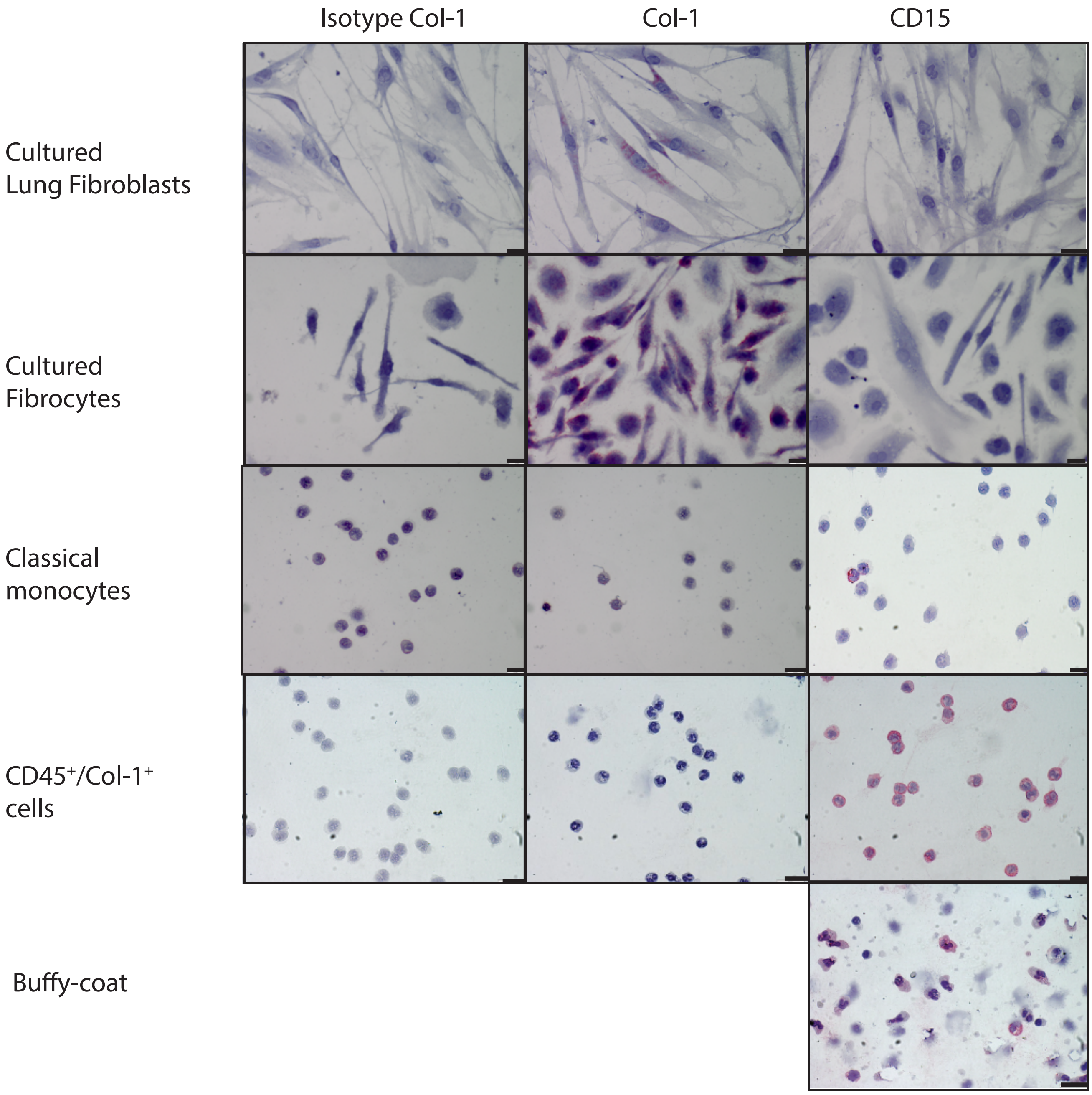
Isotype Col-1
Col-1
CD15
Cultured Lung Fibroblasts
Cultured Fibrocytes
Classical monocytes
CD45⁺/Col-1⁺ cells
Buffy-coat
